# On the Acetate of Potash in Some Diseases of the Skin

**Published:** 1851-01

**Authors:** 


*On Acetate of Potash in some Diseases of the Skin.—The Philadelphia Medical Examiner* publishes the following abstract from the Edinburgh Monthly Journal:

Dr. Easton, of Glasgow, having been led, by the publication of Dr. Golding Bird's Lectures, to employ Acetate of Potash, in some diseases of the skin, has published the results at which he has arrived, in an important practical paper, in the Edinburgh Monthly Journal, for May, 1850. Several cases are given, of Psoriasis Diffusa, Eczema Impetiginodes, Eczema Rubrum, Psoriasis Palmaris, Lepra Vulgaris, &c., which were rapidly cured under the use of the Acetate of Potash, with no other assistance than occasionally the alkaline warm bath, and fomentation of the affected extremities, with blood-letting, in one febrile case, and a catharto-diuretic mixture in another. Half a drachm of the medicine, thrice daily, is the dose for an adult.

Dr. Easton considers the medicine to act: 1. As a diuretic, as its use was always followed by a great increase in the amount of urine.

2. Not only is the amount of the *water* of the urine increased, but also its solid constituents in so remarkable a degree, as to entitle it to be considered as a renal-alterative or blood-depurant, as well as a renal-hydragogue.

3. Like all the salts of the vegetable acids, it is converted in its progress through the system, into a carbonate, and to this conversion substantially its benefit is owing. But the administration of the carbonate itself does not produce the effects which follow the use of the acetate. Hence Dr. Easton infers that the physiological action and therapeutic efficacy of the acetate are connected in some manner—unknown at present—with the metamorphosis which takes place in itself.

